# Do Gut Hormones Contribute to Weight Loss and Glycaemic Outcomes after Bariatric Surgery?

**DOI:** 10.3390/nu13030762

**Published:** 2021-02-26

**Authors:** Dimitris Papamargaritis, Carel W. le Roux

**Affiliations:** 1 Diabetes Research Centre, Leicester General Hospital, University of Leicester, Leicester LE5 4PW, UK; dp421@leicester.ac.uk; 2 Diabetes Complications Research Centre, Conway Institute, University College of Dublin, Dublin 4, Ireland; 3 Diabetes Research Group, School of Biomedical Sciences, Ulster University, Coleraine BT52 1SA, UK

**Keywords:** gut hormones, bariatric surgery, GLP-1, PYY, ghrelin, Roux-en-Y gastric bypass, gastric band, sleeve gastrectomy

## Abstract

Bariatric surgery is an effective intervention for management of obesity through treating dysregulated appetite and achieving long-term weight loss maintenance. Moreover, significant changes in glucose homeostasis are observed after bariatric surgery including, in some cases, type 2 diabetes remission from the early postoperative period and postprandial hypoglycaemia. Levels of a number of gut hormones are dramatically increased from the early period after Roux-en-Y gastric bypass and sleeve gastrectomy—the two most commonly performed bariatric procedures—and they have been suggested as important mediators of the observed changes in eating behaviour and glucose homeostasis postoperatively. In this review, we summarise the current evidence from human studies on the alterations of gut hormones after bariatric surgery and their impact on clinical outcomes postoperatively. Studies which assess the role of gut hormones after bariatric surgery on food intake, hunger, satiety and glucose homeostasis through octreotide use (a non-specific inhibitor of gut hormone secretion) as well as with exendin 9–39 (a specific glucagon-like peptide-1 receptor antagonist) are reviewed. The potential use of gut hormones as biomarkers of successful outcomes of bariatric surgery is also evaluated.

## 1. Introduction

Obesity is a complex, chronic, progressive and relapsing disease which affects currently approximately 650 million adults worldwide [1,2]. Complications of obesity include type 2 diabetes mellitus (T2D), cancer, sleep apnoea, cardiovascular, musculoskeletal, reproductive and psychological morbidities [3]. The cornerstone of prevention and treatment of obesity is behavioural changes together with diet and exercise (lifestyle changes) [4]. However, lifestyle approaches for treatment of severe obesity and its associated complications often do not achieve enough weight loss to reverse complications and weight loss maintenance remains a major challenge [5,6,7]. Current available pharmacotherapy in combination with the lifestyle changes can add a further weight loss of 2.6–8.8% [8] and help with weight maintenance [7,9], but newer medications promise more weight loss in the long term [10,11]. Even so, bariatric surgery remains a valuable tool for weight loss, leading to improvements in health, functionality and quality of life [12,13,14,15], especially for those that lifestyle- and medication-based approaches have proven ineffective to achieve and maintain clinically significant weight loss.

Currently, around 685,000 weight loss operations are performed every year worldwide [16] and the three most commonly performed procedures are the Roux-en-Y gastric bypass (RYGB), the sleeve gastrectomy (SG) and the adjustable gastric band (AGB) [16,17].

Bariatric surgery achieves successful weight loss and weight maintenance long-term as a result of reduced caloric intake postoperatively due to decreased hunger and increased satiety [18,19,20,21,22]. The changes in satiety are profound from the early postoperative period after RYGB and SG and, in some cases, there are also alterations in food preferences after these procedures [23,24]. These findings are different to what is observed after low-calorie diet, which can be very effective in the induction of weight loss, however, the vast majority of people fail to maintain the achieved weight loss in the long-term [6,7,25,26]. On a low-calorie diet, people usually report an increase in hunger, a decrease in satiety and an increased desire to eat [18,27,28] and this is likely due to robust compensatory processes that resist a drift of body fat stores below an established “set point” [29,30]. The alterations in gut hormone levels after diet-induced weight loss have been suggested as an important mediator of eating behaviours favouring weight regain in the long term [27,31]. More specifically, ghrelin, a hunger hormone, is increased after diet-induced weight loss when postprandial levels of satiety hormones are decreased [27,31].

In contrast, RYGB and SG lead to increased postprandial secretion of satiety hormones including glucagon-like peptide-1 (GLP-1), peptide YY (PYY), and oxyntomodulin (OXM) [23,32,33,34]. These changes in gut hormone secretion from the early period after RYGB and SG have been suggested as potential mediators of the increased satiety and reduced hunger postoperatively, indicating that bariatric surgery may address the dysfunctional appetite regulation in people with obesity. Moreover, significant improvements in glucose homeostasis and even remission of T2D occurs within days after bariatric surgery [35,36,37] due to well described changes in insulin secretion and sensitivity from the early postoperative period [38,39,40]. The known effect of GLP-1 on insulin secretion [41,42] combined with the elevated GLP-1 levels postoperatively further increases the interest about the gut hormones as potential mediators of these effects.

In this review, we will evaluate the currently available evidence from human studies on:

(a) The changes in gut hormones after bariatric surgery (Section 2);
(b) The role of gut hormones on weight loss outcomes after bariatric surgery (Section 3)—more specifically we will review their role as (i) biomarkers of successful weight loss postoperatively (Section 3.1 and Section 3.2) and as (ii) mediators of the postoperative changes in food intake and appetite (Section 3.3);
(c) The role of GLP-1 after bariatric surgery on glycaemic outcomes (Section 4)—we will evaluate the role of GLP-1 as mediator (i) of type 2 diabetes improvement/remission (Section 4.1) and (ii) of postprandial hyperinsulinaemic hypoglycaemia (PHH) (Section 4.2).

**Figure 1:**
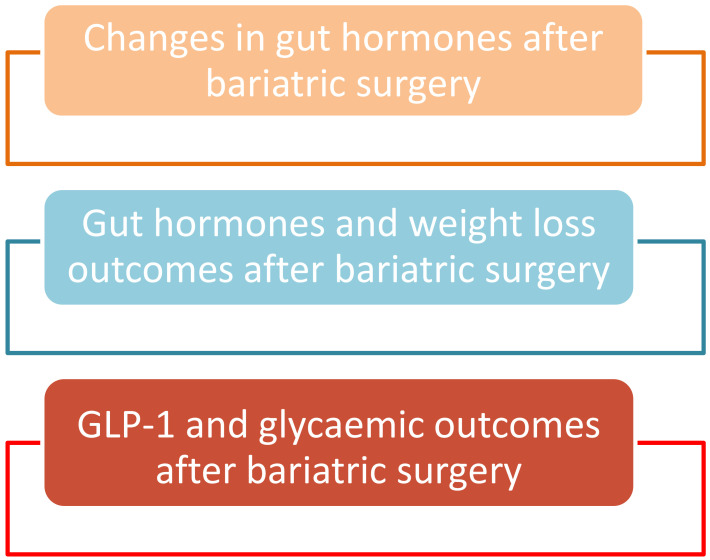
Structure of the review article.

Figure 1 demonstrates the three main sections of this review article for convenience of the readers. The focus for the sections exploring the role of gut hormones as mediators of weight loss and glycaemic outcomes after bariatric surgery will be on studies using either octreotide (a nonspecific inhibitor of gut hormones secretion) or exendin 9–39 (Ex-9, a specific GLP-1 antagonist).

## 2. Changes in Gut Hormones after Bariatric Surgery

Changes in gut hormone levels—especially those secreted from the L-cells with highest density in the ileum (such as GLP-1, PYY, OXM and glicentin)—have been consistently seen from the early postoperative period after RYGB and SG (Table 1). In contrast, gut hormone levels do not significantly change after AGB (Table 1).

### 2.1. Potential Explanations for the Increased Postprandial Gut Hormone Secretion from the Distal Gut after RYGB and SG

In people with severe obesity, gastric emptying and small intestinal transit time are slower compared to lean people and are associated with reduction in postprandial glucose absorption and glycaemic excursions as well as with reduced postprandial rise in GLP-1 and glucose-dependent insulinotropic polypetide levels (GIP) [43].

After RYGB, the rapid gastric emptying of both solids and liquids [44,45,46,47] and the bypass of the stomach and duodenum postoperatively result in accelerated nutrient delivery and absorption to the distal part of the gut [45] and subsequently to increased postprandial secretion of gut hormones [33,45,48,49]. After SG, there is increased gastric pressure [50,51], accelerated gastric emptying of nutrients [45,52,53] and accelerated small bowel transit [54] to induce an early and prolonged secretion of L-cell hormones from the intestine. In contrast, AGB does not alter the rate of gastric emptying for solids or liquids [55,56] and this is probably the reason that gut hormone secretion from the distal part of the gut is not significantly altered post-AGB [56].

There are differences between RYGB and SG on macronutrient absorption—glucose and protein absorption are accelerated after RYGB [57,58], when after SG glucose absorption is increased (but less compared to RYGB) and protein absorption is not modified [45]. These differences may account for the different hormonal profiles observed between the two procedures [34,45].

The nutrient absorption from the gut appears to be more important for the gut hormone secretion compared to the exposure of the gut to nutrients [59,60]. Further evidence on the importance of glucose absorption on GLP-1 secretion after RYGB comes from a study where sodium glucose co-transporters-1 (SGLT-1), a major mechanism of dietary glucose absorption from the gastrointestinal tract, were blocked in people who have undergone RYGB through canagliflozin, a dual SGLT-1/SGLT-2 inhibitor [61]. The study found that indeed dual SGLT-1/SGLT-2 inhibition could reduce glucose absorption and subsequently reduce peak GLP-1 levels and insulin secretion after RYGB [61].

High gastric emptying rates after RYGB for prolonged periods may also induce adaptive changes, such as an increase in enteroendocrine cell number and surface area [62,63]. Experiments with gastrostomy feeding and reversal of RYGB demonstrate acute reversal of excessive gut hormone secretion immediately after rerouting of nutrients to the stomach or after reversal of RYGB [64,65]. This supports the concept that the rapid delivery and absorption of the nutrients at the distal part of the gut constitutes a more potent stimulus for secretion of gut hormones after RYGB compared to the changes in the enteroendocrine cells.

Other mechanisms, such as changes in bile acid flow and bile acid plasma levels, may also play a role in gut hormone secretion after bariatric surgery, especially after RYGB [66,67,68]. However, further studies are required to define the role of bile acids on gut hormone secretion after bariatric surgery, particularly as evidence on changes of bile acid levels after SG is conflicting [69,70,71,72,73] and the time course of bile acid changes after RYGB is unclear [70,72,73,74].

### 2.2. Ghrelin

Ghrelin is mainly produced from the X/A like cells of the stomach and to a lesser degree from the small intestine. Ghrelin is considered to be most active in its acylated form, and is a known orexigenic hormone which stimulates appetite and food intake [75]. Furthermore, ghrelin inhibits insulin secretion in healthy people [75,76,77]. Plasma ghrelin levels are typically increased during prolonged fasting and suppressed immediately after food intake [75,78,79]. In states of increased adiposity, lower fasting plasma ghrelin levels are observed, coupled with blunted postprandial ghrelin suppression [80,81].

Changes in ghrelin levels after bariatric surgery are presented in Table 1 [33,34,82,83,84,85,86,87,88,89,90,91,92,93,94]. It is of note that during the first months after RYGB, fasting and postprandial ghrelin levels are decreased, but over the first postoperative year, ghrelin levels after RYGB gradually increase [34,86,87,88,89,90].

### 2.3. GIP

GIP is secreted from K cells found throughout the small intestine, but in highest proportion in the duodenum and jejunum. GIP increases insulin secretion following ingestion of oral glucose in healthy volunteers [41]. On the other hand, in people with T2D, the insulinotropic capacity of exogenous GIP is markedly attenuated, but it appears to be improved after near-normalisation of glycaemic control [41,95,96].

The changes in postprandial levels of GIP after RYGB and SG are inconsistent [33,48,83,97,98,99,100,101] (Table 1). On the other hand, GIP levels after AGB appear unchanged [33,56,102,103,104] (Table 1).

### 2.4. GLP-1

Glucagon-like peptide 1 (GLP-1) is secreted from L cells which predominate in the distal ileum and colon. GLP-1 stimulates insulin release in response to nutrient ingestion and reduces blood glucose levels in a glucose-dependent manner [41,105]. GLP-1 is rapidly inactivated by the enzyme dipeptidyl-peptidase-4 (DPP-4) [106]. Additionally, endogenous GLP-1 inhibits gastric emptying, inhibits glucagon secretion and has centrally mediated effects upon appetite [105,107].

Postprandial levels of GLP-1 are significantly elevated after RYGB and SG [32,33,34,45,83,85,90], but they remain stable after AGB [56,89,91,94] (Table 1). The increase in postprandial GLP-1 levels after RYGB is more profound compared to SG in some studies [34,45], but not in all [90,108].

### 2.5. PYY

PYY is a peptide secreted from intestinal endocrine L-cells of the distal gut following food ingestion along with GLP-1 [82,109]. Following cleavage in the circulation by the enzyme DPP-4, PYY 1–36 is converted to PYY 3–36 which is considered to promote satiety [81]. PYY is released postprandially in proportion to the calories ingested [110] and it increases satiety, reduces food intake, delays gastric emptying and reduces postprandial insulin secretion [111,112,113].

Alterations in PYY levels after bariatric surgery [21,32,34,45,83,85,89,91,94,108,114] are described in Table 1. Postprandial PYY levels are elevated after RYGB and SG, with more potent changes after RYGB [34,91,108,114].

### 2.6. Oxyntomodulin

Oxyntomodulin (OXM) is a peptide hormone which is structurally similar to glucagon and is produced by the L-cells of the gut [81,115]. OXM is a weak dual agonist of GLP-1 receptor and glucagon receptor [116]. A specific receptor for mediating the effect of OXM has not yet been identified in humans. Exogenous administration of OXM reduces food intake and increases energy expenditure in humans [117,118]. Few studies have assessed the changes in oxyntomodulin levels after bariatric surgery [23,33,97,119] and their results are presented in Table 1.

### 2.7. Glicentin

Glicentin contains the entire sequences of oxyntomodulin (and hence glucagon) and glicentin-related pancreatic peptide [120] and it is cleaved from the proglucagon prohormone in the L-cell. It is unclear whether glicentin is a metabolically inert by-product of proglucagon processing, which is co-secreted from the L-cell, or has a distinct function [120,121]. Currently, there is no known glicentin receptor in humans. Limited data is also available on the changes of glicentin levels after bariatric surgery [23,33,97], however postprandial glicentin levels appear to be elevated after RYGB (Table 1).

**Table 1:** Changes in gut hormones after the most commonly performed bariatric procedures.

H	RYGB	SG	AGB
Ghrelin	Fasting ↓ [85,87,88,90] or ↔ [33,34,48,88,89,91,92.94,108,114] or ↑ [86,87,88,91,108] Postprandial ↓ [48,85,90] or ↔ [33,34,48,89,94,108,114] or ↑ [89,91]	Fasting ↓↓ [23,33,34,83−85,90,93,108,114] Postprandial ↓ [23,33,85,90,114]	Fasting ↔ [33,84,89,91,94,104] or ↑ [84,91−93] Postprandial ↔ [9,91,94] or ↑ [91,94]
GIP	Fasting ↔ [33,97,98,99,101,103] Postprandial ↓ [33,103] or ↔ [48,97,101] or ↑ [98,101]	Fasting ↔ [33,83,104] Postprandial ↓ [33,104] or ↔ [105] or↑ [104]	Fasting ↔ [33,56,103,104] Postprandial ↔ [56,103]
GLP-1	Fasting ↔ [23,33,34,48,85,91,94,97−99,108] Postprandial ↑↑ [23,34,48,89,90,91,94,97−99,101,114]	Fasting ↔ [23,32−34,85,100,101,108,114] Postprandial ↑ [23,32−34,83,85,100,101]	Fasting ↔ [33,56,89,91,94,103,104] Postprandial ↔ [56,89,91,94,103]
PYY	Fasting ↔ [21,23,34,89,94,108,114] or ↑ [33,91,94,108] Postprandial ↑↑ [21,23,33,34,85,89,90,91,94,108,114]	Fasting ↔ [21,23,32−34,108,114] Postprandial ↑ [21,32−34,83,90,108,114]	Fasting ↔ [33,89,91,94] Postprandial ↔ [89,91,94] or ↑ [91,94]
OXM	Fasting ↔ [23,33,97,119] Postprandial ↑↑ [23,33,97,119]	Fasting ↔ [23,33] Postprandial ↔ [23] or ↑ [33]	Fasting ↔ [33] Postprandial NA
Glicentin	Fasting ↔ [33,97] or ↑ [23] Postprandial ↑↑ [23,33,97]	Fasting ↔ [23] or ↑ [33] Postprandial ↔ [23] or ↑ [33]	Fasting ↔ [33] Postprandial NA

RYGB: Roux-en-y gastric bypass, SG: Sleeve gastrectomy, AGB: Adjustable gastric band, GLP-1: Glucagon Like Peptide-1, PYY: Peptide YY, GIP: Glucose-dependent Insulinotropic Polypeptide, OXM: Oxyntomodulin, ↓ reduced compared to preoperatively, ↔ no change compared to preoperatively, ↑ increased compared to preoperatively, ↓↓ very reduced compared to preoperatively, ↑↑ very increased compared to preoperatively, NA: limited/no data available. Referenced studies are presented in brackets.

## 3. The Role of Gut Hormones on Weight Outcomes after Bariatric Surgery

### 3.1. Gut Hormone Levels as Predictors of Weight Loss before and after Bariatric Surgery

Werling et al. [122] reported that preoperative responses of GLP-1 and PYY to a mixed meal do not correlate with postoperative weight loss after RYGB surgery.

After RYGB and SG, the postprandial increase in oxyntomodulin and glicentin levels [% change of Area Under the Curve (AUC)]) during the first three to six months predict successful weight loss at twelve to eighteen months postoperatively [23,33] and it was also associated with favourable changes in eating behaviour [23]. The increase in glicentin levels was the strongest hormonal predictor of weight loss (was able to explain 22% of variation in weight loss at eighteen months [23]), but combining multiple gut hormones (including PYY, ghrelin, GLP-1, oxyntomodulin and glicentin) not surprisingly increased further the predictive power for postoperative weight loss, suggesting synergistic effects of gut hormones for weight loss after bariatric surgery [23]. These results may reflect that glicentin, because of its longer half-life, is probably the best marker for the secretion of proglucagon-derived hormones with already established effects on food intake and weight loss (such as GLP-1) rather than being indicative of a regulatory role for glicentin on food choice and weight loss [23]. Therefore, early postprandial responses of gut hormones, including glicentin, may be early markers of postoperative weight loss.

### 3.2. Gut Hormone Levels in Poor and Good Responders after Bariatric Surgery

As discussed, multiple satiety gut hormones are elevated after RYGB and SG, but is there a difference in gut hormone levels between “good” responders and “poor” responders to bariatric surgery? Almost all the studies which have addressed this question have been performed after RYGB. Despite the different definitions for “good” and “poor” responders between the studies, people with “poor” postoperative weight loss after RYGB have elevated ghrelin levels and reduced secretion of satiety gut hormones (mainly GLP-1 levels and in some cases PYY levels) compared to “good” responders to the operation [19,123,124,125]. Moreover, suppression of hunger was more pronounced in “good” responders to RYGB after a standardized meal compared to “poor” responders [19,125].

“Poor” responders to bariatric surgery can be further categorized as primary “poor” responders (people who have undergone bariatric surgery and achieved suboptimal maximum weight loss compared to the expected) and secondary “poor” responders (people who achieved good maximum weight loss, but then experienced significant weight regain). There is a lack of standardisation in definitions for primary and secondary “poor” responders and “good” responders [126,127]. The majority of people who are “poor” responders to bariatric surgery are secondary “poor” responders, with a 5% of bariatric surgery population being primary “poor” responders [126]. There may be a number of differences in physiology between primary and secondary “poor” responders compared to “good” responders to bariatric surgery.

De Hollanda et al. [124] evaluated whether there is a difference in gut hormones between people who are secondary “poor” responders to RYGB (*n* = 22) compared to “good” responders to RYGB (*n* = 32). They reported that secondary “poor” responders have lower GLP-1 and PYY levels and less suppression of ghrelin following a mixed meal test than “good” responders. Furthermore, ad libitum food intake (both absolute and body weight adjusted food intake) was increased in secondary “poor” responders compared to “good” responders to RYGB.

The main limitation in studies investigating differences between “good” and “poor” responders after bariatric surgery is the cross-sectional design. Thus, it is not possible to know whether the observed differences in gut hormones between “good” and “poor” responders were actually preceded the weight loss/weight gain postoperatively or are merely a consequence of the difference in body weight at the time of the study.

### 3.3. Gut Hormones and Their Role in Appetite and Food Intake after Bariatric Surgery

The increased levels of postprandial secretion of gut hormones (GLP-1, PYY) and their known role in food intake and appetite have predictably led to the hypothesis that changes in gut hormones are contributed to the observed changes in appetite and food intake after RYGB and SG. To demonstrate causality for the effect of gut hormones on satiety and food intake after bariatric surgery, a somatostatin analogue (octreotide) has been used to non-specifically attenuate the response of postprandial gut hormones (Table 2). The first evidence to strongly suggest a role for the exaggerated response of gut hormones was provided when increased food intake and reduced satiety was shown in patients after RYGB who received octreotide compared to saline (placebo) within a randomized controlled trial [19]. In contrast, patients who had undergone AGB (an operation with minimal effect on gut hormones), did not exhibit changes in appetite or food intake with octreotide administration [19].

De Hollanda et al. [124] also found that octreotide suppressed gut hormone secretion, increased food intake and suppressed satiety after RYGB. However, the observed changes in food intake and satiety were comparable in secondary “poor” responders and “good” responders to RYGB, suggesting that the role of gut hormones in the weight regain in secondary “poor” responders may be limited.

In a recently presented conference abstract, Bojsen-Moller et al. [128] reported that in primary “poor” responders to RYGB, octreotide attenuates gut hormone secretion but does not affect ad libitum food intake. In contrast, in “good” responders to the RYGB, there was an increase of 23% in ad libitum food intake with octreotide compared to placebo. Interestingly, in this study there was no significant difference in postprandial gut hormone levels (GLP-1, PYY or post-meal suppression of ghrelin) between primary “poor” responders and “good” responders. These observations suggest that impaired regulation of food intake by gut hormones may contribute to a primary “poor” response to RYGB.

As octreotide has non-specific effects to block gut hormone secretion, it is difficult to identify the main gut hormones contributing to food intake and appetite changes after RYGB. Moreover, octreotide has an inhibitory effect on gastrointestinal motility [129], which may improve postprandial symptoms and affect further the appetite postoperatively.

In an attempt to isolate the particular role of GLP-1 on inhibition of food intake after RYGB, Svane et al. [130] used the specific GLP-1 receptor antagonist Ex-9. Preoperatively, Ex-9 was associated with a 35% increased food intake in patients with obesity and T2D prior to RYGB compared to placebo. After RYGB, while food intake was less than preoperatively during Ex-9 and saline administration, there was no difference in food intake between Ex-9 and placebo. However, after administration of Ex-9, the already elevated postoperative GLP-1 and PYY levels were further increased. The increase in PYY levels was interpreted to indicate that while an inhibitory effect of GLP-1 on food intake may have been removed with the antagonist, at the same time an even greater PYY response occurs, which would favour inhibition of food intake so that the influence of Ex-9 on food intake after RYGB is neutral [130,131].

A subsequent study accordingly tried to block both GLP-1 and PYY actions [130]. Ex-9 was used to block GLP-1 actions, but as there is no available antagonist of PYY for use in humans, a DPP-4 inhibitor was used in order to inhibit the conversion of PYY 1–36 to PYY 3–36, which appears to be the actual satiety-promoting form of the hormone. In a crossover study patients after RYGB received placebo, Ex-9, sitagliptin (a DPP-4 inhibitor), and a combination of Ex-9 and sitagliptin. GLP-1 and PYY 3–36 levels were increased during placebo, and further increase following Ex-9 administration was observed as expected. The DPP-4 inhibitor caused further increases in active GLP-1 levels, but almost abolished the PYY 3–36 responses to food intake. The Ex-9 plus DPP-4 inhibitor in combination were associated with a significant increase of 20% in food intake [130,131]. These observations strongly support the concept that GLP-1 and PYY 3–36 are involved synergistically in the inhibition of appetite and food intake following RYGB.

**Table 2:** Studies using octreotide to assess food intake and satiety after bariatric surgery.

Author	Groups	No (% F)	Octreotide Dose/Saline	Meal	Age (years)	BMI Preop (kg/m^2^)	BMI at Assessment (kg/m^2^)	Time of Assessment (postoperative)	Food Intake with Octreotide vs. Placebo	Satiety/Fullness with Octreotide vs. Placebo
Le Roux 2007 [19]	RYGB AGB	7 (NR) 6 (NR)	100 mcg octreotide/ 1 mL saline	Ad libitum meal 60 min after octreotide/saline	43 ± 4.5 41.1 ± 5.6	44.5 ± 2.9 41.9 ± 7.5	33.2 ± 1.9 29.6 ± 1.5	9.5 ± 1.5 months 17.0 ± 1.4 months	↑^NC^ RYGB ↔ AGB	↑^NC^ RYGB (Fullness) ↔ AGB (Fullness)
De Hollanda 2015 [124]	RYGB, secondary “poor” responders (EWL% <50%)	19 (68.4%)	100 mcg octreotide/ 1 mL saline	Ad libitum meal 60 min after octrotide/saline	43.9 ± 10.3	46.9 ± 5.0	39.9 ± 4.0	6.5 ± 1.1 years	+53.7% (↑^ND^) secondary “poor” responders	↓(satiety) in secondary “poor” responders
	RYGB, “good” responders (EWL >50%)	23 (78.3%)			42.1 ± 10	45.6 ± 5.6	28.7 ± 3.3	6.0 ± 2.1 years	+47.3% (↑) “good” responders	↓ (satiety) in “good” responders
Bojsen-Moller 2020 [128] (abstract)	RYGB, primary “poor” responders (EBLmax <50%)	20 (100%)	1 mcg/kg octreotide (max 100 mcg)/saline	Standardised MMTT 30 min after octreotide/saline and then ad libitum meal at 270 min	51 ± 9	43.1 ± 4.0	40 ± 4.1	4.8 ± 2.0 years	−0.5% (↔) primary “poor” responders	NR
	RYGB, “good” responders (EBLmax >60%)	20 (100%)			51 ± 9	43.0 ± 3.6	29.2 ± 3.3	4.8 ± 1.4 years	+23% (↑*) “good” responders	NR

Roux-en-Y gastric bypass, EWL%: Excess Weight Loss, EBLmax: Maximum Excess BMI Loss, F: Female, MMTT: Mixed Meal Tolerance Test, NR: Not Reported, ↑: increased with octreotide vs. placebo, ↔: no change with octreotide vs. placebo, ↓: reduced with octreotide vs. placebo,*: significant difference between study groups on the outcome change with octreotide vs. placebo, ^NC^: no comparison performed between study groups on the outcome change with octreotide vs. placebo, ^ND^: no difference between study groups on the outcome change with octreotide vs. placebo.

## 4. The Role of GLP-1 on Glycaemic Outcomes after Bariatric Surgery

### 4.1. GLP-1 as a Mediator of the Improvement in Glucose Homeostasis in People with Type 2 Diabetes after Bariatric Surgery

Yoshino et al. assessed the changes in glucose homeostasis before and after matched weight loss induced by RYGB (*n* = 22) or diet alone (*n* = 22) in people with obesity and T2D [132]. After 18% weight loss, they found similar improvements in insulin sensitivity and beta cell function with RYGB and diet, suggesting that weight loss is the main driver for the observed improvements in glycaemic parameters after RYGB [132]. However, after bariatric surgery, the improvement in glucose homeostasis for people with T2D is observed from the first postoperative days, supporting the existence of weight loss independent mechanisms [36,133].

During the first postoperative weeks, the improvement in hepatic insulin sensitivity and insulin clearance due to calorie restriction and reduction in liver fat are contributed to the improvement in glucose homeostasis [36,39,134]. Additionally, the existence of RYGB mechanisms relevant to improvement in postprandial glucose metabolism which are independent to weight loss and calorie restriction is supported by the example of a patient with insulin-treated T2D who had a gastrostomy tube inserted after RYGB [135]. Five weeks after RYGB, this patient was given identical meals orally or through the gastrostomy tube on 2 consecutive days. On the day of oral feeding (200 mL of liquid meal, 300 kcal, consumed over 10 min), he had normal glucose tolerance with large GLP-1 and insulin responses; on the day of gastrostomy feeding (same meal, given over 10 min), he had glucose levels consistent with T2D and low GLP-1 and insulin levels. This was later confirmed in a larger cohort of patients [65]. These observations suggest an effect of RYGB on insulin secretion and beta cell function which can be activated by diverting food via the duodenum and it is independent of weight loss and calorie restriction.

Indeed, immediately after RYGB, beta cell function in response to a meal improves in subjects with T2D accompanied by an increased postprandial GLP-1 secretion [133,136,137,138]. Studies with Ex-9, have demonstrated causality between the increased GLP-1 secretion and the increased postprandial insulin secretion after RYGB in people with T2D (Table 3). In every study where people with T2D preoperatively were given Ex-9 vs. placebo after RYGB, at any postoperative time point, postprandial insulin secretion (whether measured as insulin, c-peptide or insulin secretion rate) was significantly reduced following Ex-9 administration and compared to the non-operated control population [37,139,140,141] (Table 3). Similar findings have been reported in people with T2D remission two years after SG [142] and in people without T2D who have undergone RYBG or SG [142,143,144,145,146]. These findings confirm that postprandial GLP-1 action is key mediator of postprandial insulin secretion after RYGB and SG; however, whether this increased insulin secretion is fundamental to the improvement in postprandial glucose levels during the early postoperative period in people with T2D is more complex, as multiple other factors may contribute to glucose homeostasis. These factors include the improved hepatic insulin sensitivity and insulin clearance due to caloric restriction during the first weeks postoperatively and, subsequently, on the gradual improvement in peripheral insulin sensitivity due to weight loss [39,131].

#### 4.1.1. Studies Using Exendin 9–39 during the Early Postoperative Period in People with Type 2 Diabetes after Bariatric Surgery

Jorgensen et al. [37] studied meal-induced responses in patients with obesity and T2D before, 1 week and 3 months after RYGB with or without simultaneous infusions of Ex-9. The Ex-9 administration impaired insulin secretion before, and particularly after, the operation; insulin secretion reverted to the preoperative values with the Ex-9 infusion at the first week and the third month postoperatively. As a result, with the administration of Ex-9 glucose tolerance worsened to preoperatively levels at the first week postoperative and was still impaired at 3 months. These findings strongly support a role of GLP-1 for improved beta cell function and improvement in glucose tolerance during the first postoperative weeks.

Another study by Shah et al. [141] assessed the role of GLP-1 action on insulin secretion and insulin clearance rate after an oral glucose tolerance test at 3 months post-RYGB, in people with T2D preoperatively (*n* = 22). Blockade of GLP-1 action with Ex-9 resulted as expected in 49% reduction in postprandial insulin levels and 51% reduction in insulin secretion rate compared to placebo as well as in 19% increase in postprandial insulin clearance rate [141]. However, this study did not have a non-surgical control group and did not report the changes in glucose levels.

On the other hand, a prospective case–control study compared the effects of Ex-9 on insulin secretion and postprandial glucose levels in 10 people with T2D after RYGB and 10 people with T2D who received an intensive lifestyle intervention after both groups have achieved 10% weight loss (12 kg) [140]. As expected, despite the comparable weight reduction between the two groups, RYGB was associated with a larger postprandial GLP-1 and insulin secretion compared to the intensive lifestyle intervention group. Ex-9 had a greater impact on insulin secretion reduction in people who have undergone RYGB than the intensive lifestyle group. Postprandial glucose levels and glucose tolerance deteriorated comparably in both RYGB and intensive lifestyle modification group during Ex-9 infusion. Therefore, this study confirms that in people with T2D preoperatively, GLP-1 action after RYGB has an important role on postoperative insulin secretion. However, the role of the excessive GLP-1 secretion after RYGB on the improvement of postprandial glucose levels and glucose tolerance after 10% weight loss may be limited compared to other factors such as the improved hepatic and peripheral insulin sensitivity, a finding in partial agreement with the study from Yoshino et al. [132]

An important factor that may contribute to the attenuated improvement in postprandial glucose levels after RYGB despite the substantial improvement in GLP-1 mediated insulin secretion is the paradoxical increase in postprandial glucagon levels after RYGB compared to the non-surgical groups, which increases further after administration of Ex-9 [37,140,147]. The underlying aetiology for the increased glucagon levels after gastrointestinal surgery is still under investigation. The glucagon concentrations after RYGB appear to follow closely the amino acid absorption and plasma amino acid concentrations [45], in agreement with recent evidence suggesting that glucagon and amino acids are linked in a mutual feedback cycle between the liver and the pancreatic a-cells [148]. Furthermore, there is evidence that part of the excessive postprandial glucagon concentration after RYGB might be gastrointestinally derived. More specifically, the expression of the glucagon gene in the small intestine appears to be increased after surgery and glucagon was identified in small intestine biopsy specimens obtained after, but not before RYGB [149]. These findings suggest that glucagon derived from small intestine enteroendocrine L cells may contribute to postprandial plasma concentrations of glucagon after RYGB [149]. Finally, accurate measurement of postprandial glucagon levels after RYGB can be challenging [150] and in some cases, the elevated plasma glucagon in people after gastrointestinal surgery may represent an assay artefact due to increased concentrations of cross-reacting proglucagon peptides [151].

Future research on the role of GLP-1 on T2D remission/improvement during the early postoperative period may also focus in people who have undergone SG. Currently there is limited number of studies with Ex-9 for people with T2D who have undergone SG (Table 3), despite that it is the most commonly performed procedure worldwide.

#### 4.1.2. Studies Using Exendin 9–39 after the Early Postoperative Period in People with Type 2 Diabetes after Bariatric Surgery

Few studies have investigated the effect of endogenous GLP-1 action on postprandial glucose levels in subjects with T2D remission who had undergone RYGB or SG at least two years before assessment, with the use of Ex-9 (Table 3) [139,142]. Jimenez et al. [139], in a cross-sectional study, assessed eight people who had undergone RYGB and achieved T2D remission and 34% weight loss at the time of assessment. A control group of seven people without diabetes and normal BMI was also recruited. In the RYGB group, insulin and C-peptide secretion decrease with Ex-9 administration, by 52% and 24%, respectively, when there was no change in insulin and C-peptide levels with Ex-9 administration in the control group. Postprandial glucose levels in the RYGB group after administration of Ex-9 increased by approximately 2 mmol/l between 90′ and 120′ post-meal which was not observed in the control group [139]. This study accordingly confirmed a role of GLP-1 for increased insulin secretion in people with T2D remission even >2 years after RYGB. Moreover, it demonstrated that GLP-1 action is contributed to the improvements in postprandial glucose levels for people who achieve T2D remission after RYGB, even if the improvement in peripheral insulin sensitivity due to substantial weight loss appears to be the main mediator of the improvement in postprandial glucose levels and T2D remission at this time.

A subsequent study from the same group assessed eight people with obesity and T2D who underwent SG more than two years ago and had achieved T2D remission. There was a control group with six BMI-matched people without T2D preoperatively who have also undergone SG at least 2 years ago and a second control group of eight people without diabetes and normal BMI [142]. Ex-9 administration was associated with impaired insulin secretion in the SG groups (the T2D remission group and the control group without T2D) compared to the non-operated control group. The blockade of GLP-1 through Ex-9 resulted in a moderate deterioration of postprandial glucose levels in all the three groups. However, postprandial glucose levels after Ex-9 were comparable between the three groups suggesting a limited role for the excess GLP-1 secretion after SG on the improvement in postprandial glucose levels in people with T2D remission after significant weight loss through SG.

An important consideration to the above studies using Ex-9 to investigate the role of GLP-1 on insulin secretion and postprandial glucose levels in people with T2D after bariatric surgery is that almost all of them have been performed in people with short duration of T2D and presumably reasonable beta cell functional capacity [37,139,140,142]. People with impaired insulin secretion and beta cell function before and after surgery are those who do not achieve T2D remission [137,152], despite that postprandial GLP-1 levels are similar between people who achieve or do not achieve T2D remission [37,139,140,142,153]. Therefore, exaggerated GLP-1 responses after RYGB and SG are probably insufficient in individuals with poor functional capacity of beta cells to secrete enough insulin and to achieve important improvement in postprandial glucose levels postoperatively [153,154].

Another limitation of studies assessing the impact of Ex-9 on glucose homeostasis after bariatric surgery is the small number of participants (the majority of studies had less than 10 participants, Table 3) and therefore the results should be interpreted with caution. Other challenges regarding the interpretation of the results include the different amount of intravenous Ex-9 administered at different studies (Table 3), as well as the increased glucagon levels after Ex-9 administration in some cases [155], which as discussed before, may affect the postprandial glucose levels. Finally, the increased levels of PYY and GLP-1 after Ex-9 administration [130] may suggest that Ex-9 interfere with intestinal endocrine feedback loops [155] which may also affect the glucose levels.

Between the other gut hormones (except of GLP-1) which may contribute to changes in glucose homeostasis after bariatric surgery, GIP is of interest due to its known insulinotropic effect. Data on GIP action after bariatric surgery is limited and GIP levels are inconsistent after RYGB and SG. There are no studies with specific GIP antagonists to assess the role of GIP on glucose homeostasis after RYGB or SG. To determine whether GIP is important after RYGB, patients without diabetes who underwent RYGB were given the DPP-4 inhibitor sitagliptin to increase the bioavailability of both GLP-1 and GIP [146,156]. They then received Ex-9 (in combination with sitagliptin) in order to block GLP-1 action and consequently, isolate the impact of GIP signalling on glucose tolerance. Interestingly, sitagliptin did not improve glucose tolerance or *beta* cell function when GLP-1 receptor signalling was blocked [146]. In contrast, patients with T2D who have not undergone bariatric procedures fully responded to the DPP-4 inhibitor with improved glucose tolerance and insulin secretion, when DPP-4 inhibitor was combined with Ex-9 [156,157]. Together these data may suggest that RYGB shifts the balance of the incretin effect towards GLP-1 and away from GIP [156]. Nevertheless, a specific GIP antagonist for human use is currently available and its use in future studies may help us understand better the role of GIP as well as the role of the combined GIP/GLP-1 action on glucose homeostasis and diabetes remission after bariatric surgery [155].

### 4.2. GLP-1 and Postprandial Hypoglycaemia after Bariatric Surgery

Postprandial hyperinsulinaemic hypoglycaemia (PHH) is a well described condition after RYGB and SG, which is associated with reduced quality of life, high degree of functional disability (inability to work, drive and care for others) and weight regain [158,159,160]. The incidence of PHH ranges between 17–75% after RYGB and SG, depending on the definition of hypoglycaemia, the population studied and the diagnostic tool used to assess hypoglycaemia [161,162,163,164,165,166,167,168]. The vast majority (around 80%) of hypoglycaemic episodes after bariatric surgery detected with continuous glucose monitoring (CGM) are asymptomatic [162], however, data on self-reported hypoglycaemia symptoms in a general population after RYGB and SG suggests that the prevalence of severe symptomatic hypoglycaemia (defined as self-reported severe symptoms of hypoglycaemia in Edinburgh hypoglycaemia scale, hypoglycaemic episodes required assistance from others, episodes of syncope, seizure or medically confirmed hypoglycaemia) is 11.6% [164]. The incidence of hypoglycaemia seems to be comparable between RYGB and SG [163], but RYGB is associated with more severe hypoglycaemic episodes and symptoms [163,166]. RYGB is also associated with higher risk of hospitalisation due to symptomatic hypoglycaemia compared to a control population, but the actual proportion of RYGB patients presented to hospital with hypoglycaemia was low (0.2%) [169]. Currently, the treatment options for PHH after bariatric surgery are limited. Patients are commonly advised to follow dietary modifications of small and frequent meals with controlled portions of low glycaemic index carbohydrates [170].

The underlying pathophysiology of PHH after RYGB is poorly defined but several studies have shown that after RYGB people who experience PHH have higher postprandial peak glucose levels, higher peak insulin secretion and higher peak GLP-1 levels compared to those without PHH [171,172,173]. PHH is a condition which often affects people without T2D preoperatively and is likely to be the outcome of the altered nutrient delivery and altered glucose absorption after RYGB rather than inherent beta-cell hypertrophy or hyperfunction [64,164]. Per this theory, feeding through gastrostomy tube to the remnant stomach after RYGB or reversal of RYGB operation can lead to remission/improvement of PHH, as well as reduction in the peak glucose, GLP-1 and insulin levels [64].

Ex-9 has been used in order to demonstrate causality between the exaggerated GLP-1 secretion and the PHH [143,144,174]. Blockade of GLP-1 action through intravenous Ex-9 in individuals with PHH reduced postprandial insulin secretion by around 50–70% [143,144,175], increased the glucagon levels, increased the postprandial glucose levels (including the nadir glucose levels) and reduced the risk and symptoms of hypoglycaemia [143,144] (Table 3). Subjects with confirmed PHH after RYGB exhibited a greater glycaemic response to intravenous Ex-9 administration with a pronounced increase in postprandial glucose [144] (Table 3). In an early phase trial in people with PHH after RYGB, subcutaneous Ex-9 increased the postprandial glucose nadir by 66%, reduced peak insulin levels by 57%, and reduced neuroglycopenic symptoms by 80% [174] (Table 3). A phase 2 trial confirmed the safety, tolerability and efficacy of subcutaneous GLP-1 antagonist as a treatment for PHH [176]. More specifically, twice daily administration of subcutaneous exendin 9–39 for 3 days, effectively raised the nadir glucose levels by 39–47% and improved by 47% the symptoms suggestive of hypoglycaemia after an oral glucose tolerance test (see also Table 3) [176].

## 5. Pharmaceutical Use of Gut Hormones after Bariatric Surgery

Multiple GLP-1 receptor analogues (GLP-1 RA) are currently available for the management of obesity and T2D. The GRAVITAS (GLP-1 Receptor Agonist interVentIon for poor responders after bariAtric Surgery) randomized controlled trial [177], demonstrated that in individuals with persistent or recurrent T2D after RYGB or SG, use of 1.8 mg liraglutide once daily in combination with lifestyle advice and support for 6 months reduced HbA1c by 1.2% and led to an additional weight loss of 4.2 kg compared to placebo [177]. Moreover, in a recent retrospective analysis of 2092 patients, the use of liraglutide 3.0 mg (licensed for treatment of obesity) was evaluated in patients who have had bariatric surgery (*n* = 188), as well as non-surgical patients [178]. Weight loss achieved was approximately 6% after ≥ 16 weeks, which was comparable between the surgical and non-surgical groups, confirming the potential effectiveness of liraglutide 3.0 mg after bariatric surgery [178]. Observational studies using liraglutide 3.0 mg after bariatric surgery have reported similar results regarding weight loss [179].

These findings suggest that despite the increased endogenous postprandial GLP-1 secretion after RYGB and SG, GLP-1 RA could still be effective after bariatric surgery on improving weight loss and glycaemic control in T2D. Currently, a number of clinical trials with liraglutide 3.0 mg are taking place for treatment of “poor” responders to bariatric surgery [180] or for treatment of weight regain after bariatric surgery [181].

## 6. Conclusions

Each bariatric procedure has a unique gut hormone profile. Changes in gut hormones after RYGB have an important and synergistic role to increase satiety and reduce food intake postoperatively. The best evidence currently exists for GLP-1 and PYY 3–36 to reduce food intake after surgery. Moreover, postprandial changes in glicentin and oxyntomodulin during the early postoperative period after RYGB and SG are associated with weight loss outcomes. These hormones have the potential to be used as early markers of inadequate postoperative weight loss to identify people who may require additional postoperative supportive care.

GLP-1 contributes also to increased insulin secretion after RYGB from the early postoperative period in people with and without T2D. GLP-1 may also be pivotal to PHH after RYGB and ongoing studies are assessing the safety and efficacy of a subcutaneous GLP-1 receptor antagonist (Ex-9) as a treatment option. Interpretation of the role of enhanced GLP-1 responses to improved postprandial glucose levels in people with preoperative T2D is confounded by the effects of caloric restriction on glucose homeostasis during the early postoperative setting and the effects of weight loss in the late postoperative setting.

Based on the described changes in gut hormones after RYGB and SG and the evidence of their synergistic action in reducing food intake and appetite, combinations of gut hormone receptor agonists aiming to replicate the hormonal changes after bariatric surgery are under development and may form the next generation of treatments for obesity and T2D [182].

## Figures and Tables

**Table 3:** Studies using the GLP-1 antagonist Exendin 9–39 to assess glycaemic outcomes in people with type 2 diabetes or postprandial hypoglycaemia after bariatric surgery.

Author	Groups	No (F%)	Meal	Ex-9 Dose/Placebo	Age (years)	BMI before Intervention (Kg/m^2^)	BMI at Assessment (Kg/m^2^)	Time of Assessment	T2D Duration (years)	Glucose Parameters with Ex-9 vs. Placebo	Insulin Parameters with Ex-9 vs. Placebo	C-Peptide Parameters with Ex-9 vs. Placebo
**Studies in Population with T2D Preoperatively.**												
Jorgensen 2013 [37]	RYGB, T2D preoperatively	9 (33%)	MMTT, 300 kcal, 50% carbs, 35% fat, 15% protein	43,000 pmol/kg bolus and then 900 pmol/kg/min OR saline (IV)	50 ± 3		37.67 (mean)	1 week postop	5.7 ± 1.3	+64.6% (↑^NC^) AUC (0–240) +28.8% (↑^NC^) 2 h glucose	−22.9% (↓^NC^) AUC (0–240)	−28.4% (↓^NC^) AUC (0–240)
	RYGB, T2D preoperatively						34.03 (mean)	3 months postop		+39.2% (↔ ^NC^) AUC (0–240) +29.2% (↑^NC^) 2 h glucose	−31.7% (↔ ^NC^) AUC (0–240)	−25.5% (↓^NC^) AUC (0–240)
	Preoperative values used as control					39.2 ± 2.4		Preop		+49.7 (↑) AUC (0–240) +18% (↑) 2 h glucose	−2.9% (↔) AUC (0–240)	−2.1% (↔) AUC (0–240)
Vetter 2015 [140]	RYGB, T2D preoperatively	10 (90%)	MMTT, 240 Kcal, 55% carbs, 25% protein 20% fat	7500 pmol/kg bolus and then 750 pmol/kg/min OR saline (IV)	54 ± 6.6	43.2 ± 1.9	39.1 ± 1.4	58.9 ± 12.1 days postop	5.2 ± 3.3	+41.4% (↑^ND^) AUC (0–180)	−44.9% (↓^ND^) AUC (0–180)	−37.1% (↓*) AUC (0–180)
	Intensive lifestyle modification (ILM), T2D at baseline	10 (50%)			51.8 ± 11.6	41.8 ± 1.2	37.3 ± 1.4	85.5 ± 24.4 days post-ILM initiation	3.1 ± 2.7	+44.4% (↑) AUC (0–180)	−10% (↔) AUC (0–180)	−5.1% (↔) AUC (0–180)
Shah 2019 [141]	RYGB, T2D preoperatively	22 (91%)	75 g of oral glucose	600 pmol/kg/min OR saline (IV)	44.1 ± 8.6	42.1 ± 5.1	34.9 ± 4.6	3 months	8.26 ± 7.6	NR	−48.8% (↓^NA^) in AUC (0–180)	−51.1% (↓^NA^) in ISR AUC (0–180)
	No control group											
Jimenez 2013 [139]	RYGB, T2D remission	8 (100%)	MMTT, 398 kcal, 50% carbs, 35% fat, 15% protein	7500 pmol/kg bolus and then 750 pmol/kg/min OR saline (IV)	54.1 ± 8.4	46.8 ± 6.6	30.8 ± 4.7	NR (>24 months postop)	2.1 ± 1.1	+10.07% (↑^NC^) AUC (0–120) +NR (↑^NC^) 2 h glucose	−53.8% (↓^NC^) AUC (0–120)	−24.9% (↓^NC^) in AUC (0–120)
	Healthy controls	7 (NR)			47.0 ± 10.8	NA	21.1 ± 1.3	NA	NA	+9.3% (↑) AUC (0–120) +NR (↔) 2 h glucose	−4% (↔) in AUC (0–120)	−2.9% (↔) in AUC (0–120)
**Author**	**Groups**	**No (F%)**	**Meal**	**Ex-9 Dose/Placebo**	**Age (years)**	**BMI before Intervention (Kg/m^2^)**	**BMI at Assessment (Kg/m^2^)**	**Time of Assessment**	**T2D Duration** **(years)**	**Glucose Parameters with** **Ex-9 vs. Placebo**	**Insulin Parameters with Ex-9 vs. Placebo**	**C-Peptide Parameters with Ex-9 vs. Placebo**
Jimenez 2014 [142]	SG, T2D remission	8 (67%)	MMTT, 398 kcal, 50% carbs, 35% fat, 15% protein	7500 pmol/kg bolus and then 750 pmol/kg/min OR saline (IV)	49.8 ± 12.4	47.7 ± 5.5	32.7 ± 2.3	3.4 ± 0.9 yearspostop	2.8 ± 1.8	+12.4% (NR) in AUC (0–120) +23.7% (↑) in 2 h glucose	−18.4% (↓*) in total insulin output	−39.1% (↓) in b-cell glucose sensitivity
	SG, without T2D preop	6 (67%)			52.1 ± 13.1	44.9 ± 5.3	31.1 ± 4.2	2.9 ± 0.9 years postop	NA	+2.9% (NR) in AUC (0–120) +16.7% (↔) in 2 h glucose	−11.1% (↓*) in total insulin output	+3.3% (NR) in b-cell glucose sensitivity
	Healthy controls	8 (67%)			50 ± 13	NA	23.3 ± 2.0	NA	NA	+9.4% (NR) in AUC (0–120) +12.7% (↔) in 2 h glucose	+2.4% (↔) in total insulin output	−33.7% (NR) in b-cell glucose sensitivity
**Studies in Populations with Postprandial Hyperinsulinaemic Hypoglycaemia**												
Salehi 2014 [144]	RYGB with established PHH	9 (100%)	MMTT, 350 kcal, 57% carbs, 28% fat, 15% protein	7500 pmol/kg bolus and then 750 pmol/kg/min OR saline (IV)	44.6 ± 4.5	48 ± 2.6	30.9 ± 2.5	3.9 ± 0.5 years postop	NA	+67.3% (NR) in nadir levels +250.4% (↑*,**) AUC (0–180)	−63.3% (↓*,**) AUC (0–180)	−46.8% (↓*,**) in ISR AUC (0–180)
	RYGB without symptoms of PHH	7 (43%)			47.6 ± 3.0	55 ± 2.6	33.8 ± 3.4	3.6 ± 0.7 years postop	NA	+14.3% (NR) in nadir levels +32.1% (↔ ^ND^) AUC (0–180)	−19.2% (↔ ^ND^) AUC (0–180)	−22.4% (↔ ^ND^) in ISR AUC (0–180)
	BMI-matched controls	8 (88%)			33.1 ± 3.3	NA	32.8 ± 1.1	NA	NA	+9.8% (NR) in nadir levels +5.4% (↔) AUC (0–180)	−22.2% (↔) AUC (0–180)	−22.6% (↔) in ISR AUC (0–180)
Craig 2017 [143]	RYGB with established PHH	8 (100%)	75 g of oral glucose	7500 pmol/kg bolus and then 750 pmol/kg/min OR saline (IV)	46.4 ± 4	NR	31.2 ± 2	5 years postop	NA	+69.2% (↑) in nadir levels +21.1% (↑) AUC (0–180)	−56% (↓) in peak levels −57.1% (↓) AUC (0–180)	−51.4% (↓) AUC (0–180)
	BMI-matched controls	8 (100%)			47 ± 3	NA	31.0 ± 0	NA	NA	NA	NA	NA
Craig 2018 [174]	RYGB with established PHH	8 (100%)	75 g of oral glucose	0.13–0.38 mg/kg (subcut)	45 ± 3.8	49 ± 2.3	29 ± 1.3	6.9 years postop	NA	+66% (↑^NA^) in nadir levels +72% (↑^NA^) AUC (90–180)	−57% (↓) in peak levels −48% (↓) AUC (0–60)	−44% (↓) in peak levels −31% (↓) AUC (0–60)
	No control group											
Tan 2020 [176]	RYGB with established PHH (treated with Lyo Ex-9)	14 (100%)	75 g of oral glucose	0.05–0.46 mg/kg bd for 3 days (subcut)	45 ± 5	48 ± 3	28 ± 4	8.6 years postop	NA	+39% (↑^NA^) in nadir levels^ +79% (↑^NA^) AUC (90–180)^	−50% (↓) in peak levels^ −47% (↓) AUC (0–60)^	NA NA
	RYGB with established PHH (treated with Liq Ex-9)	5 (100%)		0.38 mg/kg bd for 3 days (subcut)	51 ± 3	50 ± 4	30 ± 4	10.2 years postop	NA	+47% (↑^NA^) in nadir levels +71% (↑) AUC (90–180)	−67% (↔) in peak levels −63% (↓) AUC (0–60)	NA NA
	No control group											
Salehi 2011 [175]	RYGB without symptoms of PHH	12 (75%)	**MMTT with clamp** MMTT, 57% carbs, 15% protein, 28% fat	7500 pmol/kg bolus and then 750 pmol/kg/min OR saline (IV)	47 ± 2	52 ± 2	33 ± 1	3.3 ± 0.3 years postop	NA	0% AUC (95–270) (**clamp study**)	−50% (↓*) AUC (95–270)	−28% (NR) AUC (95–270)
	RYGB with symptoms of PHH	12 (92%)			39 ± 2	52 ± 2	32 ± 2	3.7 ± 0.4 years postop	NA	0% AUC (95–270) (**clamp study**)	−54% (↓*) AUC (95–270)	−37% (NR) AUC (95–270)
	BMI matched controls	10 (80%)			43 ± 3		33 ± 2	NA	NA	0% AUC (95–270) (**clamp study**)	−16% (NR) AUC (95–270)	−12% (NR) AUC (95–270)

Roux-en-Y gastric bypass, T2D: Type 2 Diabetes Mellitus, F: Female, MMTT: Mixed Meal Tolerance Test, SG: Sleeve Gastrectomy, PHH: Postprandial Hyperinsulinaemic Hypoglycaemia, Ex-9: Exendin 9–39, AUC: Area Under the Curve, 2 h: 2 h, ISR: Insulin Secretion Rate, bd: twice daily, NA: Not Applicable, NR: Not Reported, ↑: increased with Ex-9 vs. placebo, ↔: no change with Ex-9 vs. placebo, ↓: reduced with Ex-9 vs. placebo,*: significant difference between surgical and non-surgical group on the outcome change with Ex-9 vs. placebo, ^NC^: no comparison performed between study groups on the outcome change with Ex-9 vs. placebo, ^ND^: no difference between surgical and non-surgical group on the outcome change with Ex-9 vs. placebo, **: significant difference between surgical groups on the outcome change with Ex-9 vs. placebo, ^ the analysis is for a subgroup of 6 participants received ≥20 mg (≥0.35 mg/kg) of subcutaneous Lyophilized (Lyo) Ex-9, Lyo: Lyophilized Ex-9, Liq: Liquid form of Ex-9, IV: intravenous, subcut: subcutaneous.
